# Co-precipitation with PVP and Agar to Improve Physicomechanical Properties of Ibuprofen

**Published:** 2013-04

**Authors:** Maryam Maghsoodi, Farhad Kiafar

**Affiliations:** 1 Drug applied Research Centre and School of Pharmacy, Tabriz University of Medical Sciences, Tabriz, Iran

**Keywords:** Agar, Co-precipitation, Crystallization, Ibuprofen, PVP

## Abstract

***Objective(s)*** *:* Ibuprofen is a problematic drug in tableting due to its viscoelastic properties. Additionally its high cohesivity results in low flowability. In this study, co-precipitation of ibuprofen with varying concentration of agar and PVP to optimize properties of Ibuprofen was carried out.

***Materials and Methods:*** Co-precipitates of ibuprofen- PVP or agar were prepared by solvent evaporation technique under vacuum condition. Differential scanning calorimetry (DSC), X -ray diffraction of powder (XRDP) and FT-IR spectroscopy were used to investigate the solid state characteristics of the co-precipitates. The dissolution behavior, flowability, particle size and compaction properties of various batches were also studied.

***Results: ***Co-precipitation of drug with agar led to a change in habit from needle to plate shape crystals, while drug –PVP co-precipitates had agglomerated structure and consisted of numerous crystals which had been aggregated together. The co-precipitates showed improved flow properties compared with ibuprofen alone. Precipitation of ibuprofen with these additives led to modification in the dissolution of the drug. Agar in 1% w/w improved slightly the dissolution rate of drug while PVP had a negative impact and led to reduction in the dissolution rate of drug to less than that of pure drug. The all obtained co-precipitates exhibited significantly improved tableting behavior compared with drug crystals alone. This may be due to this fact that, the polymer covering the drug particles increases and changes the nature of the surface area available for interparticulate bonds between particles. DSC, XRDP and FT-IR experiments showed that drug particles, in co-precipitates samples, did not undergo polymorphic modifications.

***Conclusion:*** The study highlights the influence of polymeric additives on crystallization process leading to modified performance.

## Introduction 

Direct tableting has been renewed as a preferable process by simply mixing and compressing powder to save time and cost in comparison with granule tableting. The direct compression of a powder depends on its flowability and mechanical properties (1). Some drug crystals exhibit appropriately such properties, but many materials have very poor flowability and compactibility. 

Direct tableting of latter materials has been successfully industrialized by coformulating higher amounts of additives (≥75%) (2). 

However, it is desirable to reduce the amount of additive, thus decreasing the size of the dosage form, in order to improve patient compliance and save production costs. On the other hand, the use of direct compression in the production of high –dose formulations is limited, since large quantities of excipients are ordinarily required to produce suitable tablets. To achieve this goal, the micromeritic properties such as flowability, packability, compressibility, etc. of the drug must be improved with the aid of minimum amount of additive. Ibuprofen is a high–dose nonsteroidal anti-inflammatory agent which has poor flowability and compaction characteristics owing to its needle –like (acicular) crystalline structure and viscoelastic properties, respectively (3,4). The properties of a drug can be improved by choosing suitable polymorphic form and crystal habit and by using a suitable preparation with excipients.

In the literature several methods for improving the properties of ibuprofen have been described. Usually a preparation with excipients is used to optimize the substance properties. A co-precipitationwith Eudragit S100 is described by Khan *et al* (5).

Kachrimanis *et al* (6) also described spherical crystal agglomerates obtained by crystallization by the solvent change technique in the presence of Eudragit S100. A powder with a drug-load of 90% (m/m) was obtained. Flowability and compressibility were improved because of the Eudragit S100 and the drug release was sustained. Pawar *et al* (7) described agglomerates of ibuprofen with talc prepared by crystallo-co- agglomeration technique to obtain directly compressible agglomerates of ibuprofen. Co-precipitation of drug and additives by solvent change method has been widely investigated as mentioned in above studies. In this method, there is a need for quantitative determination of additives for understanding their effects (8, 9). This is not straight forward due to the lack of UV-absorbing chromophores of the most commonly employed excipients. However, solvent evaporation method is capable of preparation of co-precipitates of drug with definite and reproducible amount of additives compared to solvent change method.

The study described in this article investigated simple method to prepare co-precipitate of ibuprofen with additives with optimized properties that are potentially suited for production tablets. Co-precipitation with different additives was carried out by the solvent evaporation technique and the properties of various co-precipitates were investigated. The other advantage of this method is that solvent evaporation technique is simple, reproducible and obtained products have high yield in comparison with methods described above.

## Materials and Methods 

Ibuprofen (Boots Limited, UK), povidon (PVP K30) (BASF, Ludwigshafen, Germany), agar and ethanol (Merck, Germany) mineral oil (Sigma Chemical Co., St. Louis, USA) were used. 

Preparation of co-precipitates:

Co-precipitation was carried out by solvent evaporation technique. 5 g ibuprofen was dissolved in 20 ml ethanol. Then 5,25 and 50 mg PVP or aqueous agar solution containing 5, 25 and 50 mg agar (10 ml) for preparation of respectively 0.1, 0.5 and 1% w/w of Drug –PVP or Drug –agar co-precipitates were added to ethanolic solution of drug. Afterwards, in order to evaporate the solvents resultant solutions stirred with a magnetic stirrer under vaccum at room temperature. Final solvent removal was done after manual gently scraping of the co-precipitate and drying in oven at 30 ^o^C overnight. The dried samples were stored in a desiccator at room temperature before use. 

*Micromeritic properties*

The particle size of crystals was measured by a microscopic method. A small amount of powder (about 20 mg) was suspended in mineral oil and the suspension was spread onto a microscope slide. A cover slip was applied, allowing the suspension to settle homogeneously between the two glass surfaces. Pictures of each powder were taken using a CCD camera (Canon digital, Japan) connected to a light microscope (Nikon Labophot, Tokyo, Japan). Feret’s diameter of at least one hundred particles was measured by the scion image analysis software via obtained photographs to determine the mean (arithmetic) particle size.

Flowability and packability of samples was assessed by determination of Carr’s Index (CI) (10, 11). The CI was calculated from the poured and tapped densities. Tapped density was determined by tapping the samples into a 25 ml measuring cylinder using a tapping machine until the volume did not change significantly (300 tap). The CI was calculated according to the following equation.

CI=(((Tapped density- Bulk density))⁄(Tapped density))×100

*Determination of ibuprofen solubility*

The solubility of the powders was determined in water, by adding excess powder in the liquid. The solution was passed through a 0.45 µ membrane filter and the amount of the drug dissolved was analyzed by UV-Spectrophotometer at 262 nm after suitable dilution (2 times) until such time as the absorbance reading of a sample was the same on three successive days. The experiments were undertaken at 25 ± 0.1°C. In order to determine the aqueous solubility of drug in presence of the additives, the same procedure was carried out, but the drug powder was dispersed in 100 ml freshly distilled water containing various concentrations of additives (12). The mean of three determinations was used to calculate the solubility of drug in the aqueous media.

*Contact angle determination*

Dry powder was compressed at 1000 kg compression force using a hydraulic press (Riken Seiki Co., Japan) with 8 mm diameter flat faced punches. A droplet of water (3 µl) was placed onto the surface of the compact and observed using a low power microscope. The contact angle was determined by measuring the tangent to the curve of the droplet on the surface of the compact (13). The contact angle θ can be calculated using the following equation: tan θ/2=H/R in which H is the drop height and R is the radius of the drop base. 

*In Vitro release studies*

The *in vitro* dissolution of ibuprofen samples was determined with a USP rotating paddle method. A suitable amount of samples (equal to 30mg ibuprofen) for sink condition were dispersed directly in dissolution vessel containing 900 ml phosphate buffer (pH 7.4) maintained at 37±0.5 °C and stirred at 50 rpm. At preset time intervals, aliquots were withdrawn and replaced by an equal volume of dissolution medium to maintain constant volume. The solution was passed through a membrane filter (0.45μm) and then the concentration of ibuprofen in solution was measured with an ultraviolet spectrophotometer (Shimadzu 120A, Japan) at a wavelength of 221.8nm. All samples analyzed in triplicate.

*Preparation and characterization of the compacts*

The crystals were directly compacted using 8 mm flat-faced punches on a hydraulic press (Riken Seiki Co., Japan). The material for each tablet was weighed (100 mg), introduced into the die and compacted at compression pressures of 25 and 50 MPa. The die wall and punch surfaces were lubricated with 1% w/w magnesium stearate in ethanol before compaction. The compacts were held under load for 30 s, ejected and stored in screw-capped bottles for 24 hr before using, to allow for possible hardening and elastic recovery. The force required to fracture the compacts on a motorized tablet hardness tester (Erweka, Germany) was measured to determine tablet crushing strength. The tensile strength of the compact was calculated using the following equation (14):

T=2F⁄πDt

in which D and t are the diameter and thickness of the compact, respectively, and F is the force fracturing the compact. Experiments were repeated five times for statistical reliability and the mean values of five determinations were reported.

*FT-IR spectroscopy*

Infrared spectra were recorded using a FT-IR spectrophotometer (M-B-100, Bomem, Canada) utilizing potassium bromide discs. Samples were prepared by gently grounding the powder with KBr. The data region was 4000-400 cm-1.

*X -ray diffraction of powder (XRDP)*

A Seimens (Model D5000, Germany) x-ray diffractometer was used at 40 kV, 30 mA and a scanning rate of 0.06º min^-1^ over a range of 2-40 2θ, using CuK_α__1_ radiation of wavelength 1.5405 Ǻ.

*Differential scanning calorimetery (DSC)*

DSC can be used to determine polymorphic composition of pharmaceutical powders when the polymorphs present different melting points. After calibration with indium a lead standards, samples of the crystals (3–5 mg) were heated (range 50–170 °C) at 10 °C/min in crimped aluminum pans under a nitrogen atmosphere. The enthalpy of fusion and melting point were automatically calculated (Shimadzu, Japan).

*Statistical evaluation of data*

Quantitative data were reported as mean ± standard deviation (SD). Statistical analysis was performed using the analysis of variance (ANOVA). Comparison between the two means was determined using the Tukey’s test with statistical significance evaluated at *P<* 0.05. Statistical analysis of the dissolution data using the percentage of drug released with time as the quantitative parameter was undertaken. 

## Results 

*Micromeritic properties*

**Figure 1:**
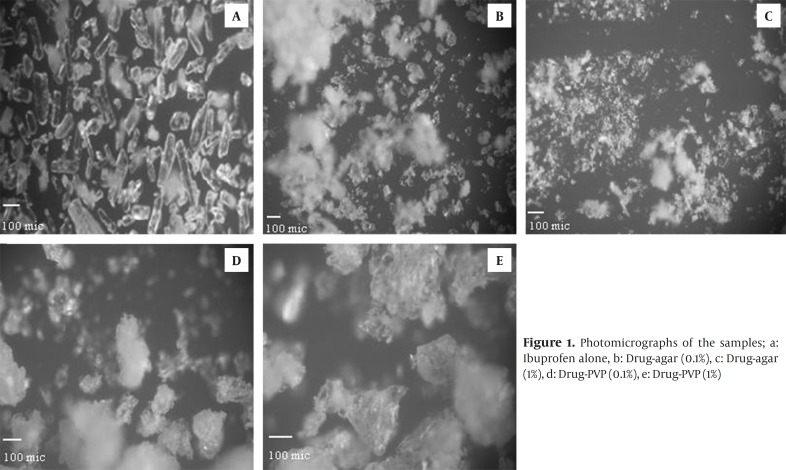
Photomicrographs of the samples; a: Ibuprofen alone, b: Drug-agar (0.1%), c: Drug-agar (1%), d: Drug-PVP (0.1%), e: Drug-PVP (1%)

The morphologic features of various crystals generated in the presence of additives were visually examined using light microscopy. Co-precipitate powder contains crystals embedded in a polymer matrix shown in the Figure1. Table 1 shows the average range particle size for crystals generated using various concentrations of additives. 

Figures. 1d and f illustrate drug particles crystallized in presence of PVP. These figures clearly indicate that the use of PVP in the crystallization medium had a major effect on the morphology of drug crystals and affect the crystal growing in a special manner. 

Table 1 shows the compressibility values (Carr Index) of ibuprofen and co-precipitates samples. The Carr index revealed that the flowability of ibuprofen was very poor (>40). 

*FT-IR, X-ray and DSC*

**Figure 2:**
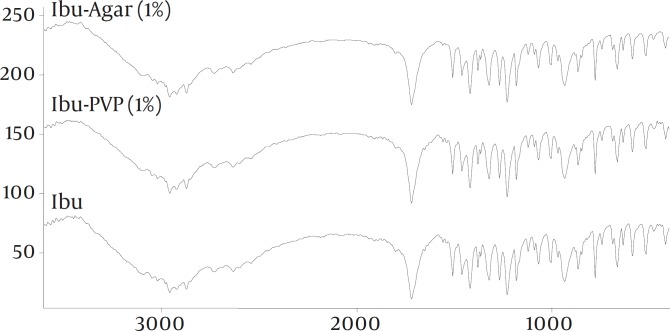
FT-IR spectra of the samples

**Figure 3:**
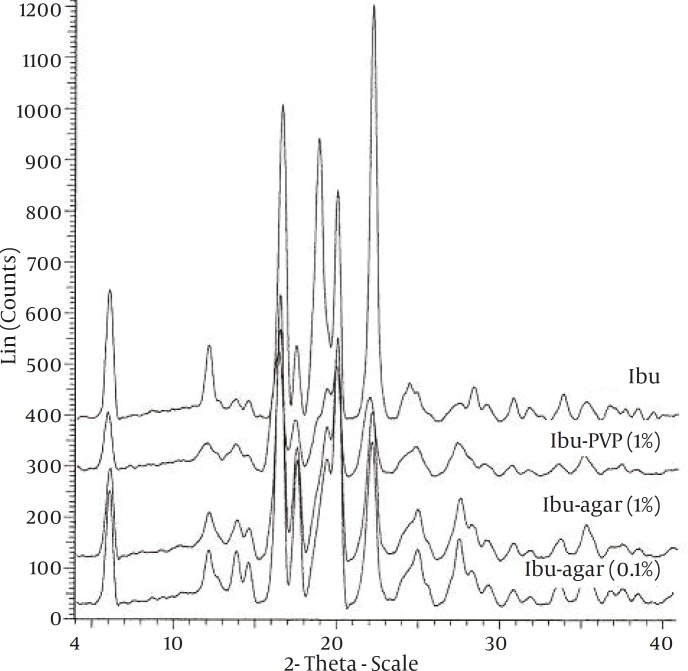
The X-ray diffraction spectra of the samples

To study the possibility of an interaction of drug with the additives in the solid state, FT-IR spectroscopy was carried out (Figure 2). The spectra of pure drug and the co-precipitates did not show any significant difference. XRPD patterns of the pure drug and co-precipitates are shown in Figure 3. The XRPD patterns of the co-precipitates did not show any significant difference with pure drug. There was no change observed in the d-spacing value of various samples. However, the relative intensities of their XRPD peaks were modified. For example, there is a peak at 19⁰ 2θ, in pure and untreated ibuprofen which almost has been vanished in co-precipitate samples.

**Figure 4:**
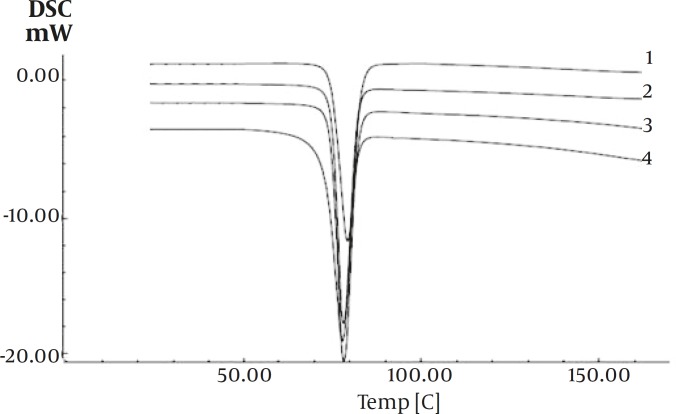
Thermograms of the samples; 1:drug –PVP (1%), 2:drug-agar (1%), 3: drug-agar (0.1%), 4: ibuprofen without additives

DSC curves of pure drug and the co-precipitated samples are shown in Figure 4. All samples showed a sharp melting point with flat baseline which indicated that no events such as hydration, solvation or polymorphic transition had occurred during crystallization of the particles. The mean values of the melting points and enthalpies of fusion for ibuprofen and the co-precipitated samples are presented in Table1. Results indicates that the melting point and enthalpies of fusion of the co-precipitated samples decreased by 0.13-1.5°C and 0.74-5.43 cal/g, as compared to ibuprofen alone. 

*Tabletability*

The effect of compression force on the tensile strength of tablets made from the samples is reported in Table 2. Compression of ibuprofen crystals at all compaction pressures produced weak compacts with low tensile strength. The compact hardness of the drug significantly increased in the presence of the additives and with increasing concentration (*P<*0.05). The compression studies showed that the tensile strength of tablets made from the co-precipitates were sensitive to compression pressures. In other words, the tensile strengths of those tablets markedly increased as the compression pressure was increased from 25 to 50 MPa. 

**Table 1:** Results of solubility, contact angle, Carr index, particle size, melting point and enthalpy of fusion for the samples (mean±SD, n=3)

samples	Solubility (µg/ml)	Contact angle (^o^)	Carr index (%)	Mean Particle size (µm)
Ibuprofen	52.1±9	58.2±5.2	40±1.2	120/20 (length/breadth)
Agar 0.1%	60.5±8	30.3±4.8	33±1.1	100/15 (length/breadth)
Agar 0.5%	63.5±6	28.4±3.6	34±0.9	50/8 (length/breadth)
Agar 1%	64.3±5	38.3±4.3	31.3±1.1	10
PVP 0.1%	58.3±8	34.3±3.9	22±0.8	110
PVP 0.5%	59.6±7	25.3±4.2	18.5±0.9	150
PVP 1%	57.8±9	28.2±3.5	24±0.9	250

**Table 2.** The tensile strength of the compacts of the samples (mean±SD, n=5)

Sample	Tensile strength(MPa)	
	Compression pressure	
	25 (MPa)	50 (MPa)
Ibuprofen	0.51±0.20	0.60±0.22
Agar 0.1%	8.86±0.81	9.7±0.61
Agar 0.5%	8.05±0.75	10.09±0.54
Agar 1%	8.19±0.65	12.52±0.78
PVP 0.1%	8.55±0.58	10.75±0.81
PVP 0.5%	10.68±0.62	13.56±0.85
PVP 1%	11.10±0.72	14.40±0.63

*Dissolution*

**Figure 5:**
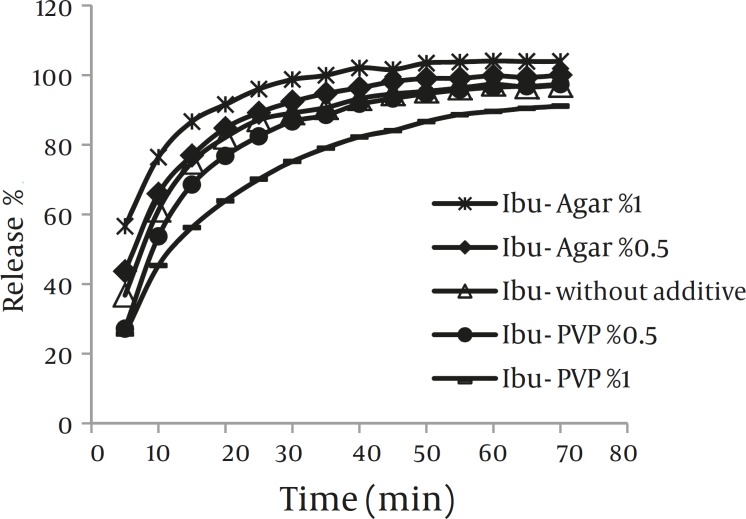
Dissolution profiles of the co-precipitated samples and ibuprofen powders (Mean±SD, n=3)

Results of the dissolution study are illustrated in Fig.5. The percentage of drug dissolved within the first 20 min was used to compare dissolution profiles of the various samples. According to this Fig, co-precipitation with agar (0.5% and 1%) produced crystals with slightly higher dissolution rate than crystals obtained without any additive. However, co-precipitation with PVP (0.5% and 1%) led to decreasing dissolution rate of drug even lower than pure ibuprofen. 

The aqueous solubilities of drug crystals alone and also in presence of additives are listed in Table 1. There were no significant differences between the solubility of these samples (*P>*0.05). Table 1 also gives the mean contact angle values of water droplets on the sample surfaces. The results for co-precipitated samples indicate better wettability (lower contact angle) by water compared to pure ibuprofen (θ= 58.2).

## Discussion

*Micromeritic properties*

It has been reported that effective additives influence the crystallization process and produce crystals of a different shape to those formed from a pure solution (15). Ibuprofen recrystallized without polymer occurs as fine micronized needles, while the recrystallized product using agar and PVP had different external appearances. Crystallization in the presence of agar led to a change in habit from needle to plates. Also an increase in the concentration of agar led to reduction in crystal size. The crystals generated in the presence of agar (1%) were very fine and in the range of 10 µm. This may be because polymers prolong supersaturation and increase the viscosity of the medium for controlling crystallization (16). Reported studies have suggested that adsorption of polymer on the surface of nuclei leads to the formation of a diffusional boundary layer, which inhibits nucleation and growth, resulting in finer crystals yield (16, 17).

Figs. 1d and f show that the majority of the crystals obtained in presence of PVP are aggregated.

PVP has been shown to be a strong crystallization inhibitor for numerous drugs including captopril and levonorgestrel (18), zolmitriptan (19), and sulfamerazine (20). When PVP was added to supersaturated solutions of sulfathiazole, the growth of the seeded crystals initially slowed down and then completely stopped. A similar effect is likely for the ibuprofen-PVP systems studied. PVP because of its strong inhibitory effect on crystal growth, doesn’t allow for construction of initial big crystals and therefore fine microcrystals were formed. Aggregation of these very small crystals of ibuprofen could be expected by their flat shape and high hydrophobicity as it has been reported by other research (21). Obviously, by increasing concentration of PVP, the inhibitory effect of PVP on crystal growth has been promoted and finer crystals with higher trend to aggregation have been produced which interpreted forming larger particles. 

In order to achieve uniformity in tablet weight, the feed crystals must flow and pack smoothly into the die cavity of the tablet machine. Therefore, in design of particles for direct compression it is essential to improve the flow and packing properties. The low values of Carr’s index for co-precipitates in comparison to drug alone indicated their better flowability and packability (10, 11). The flowability correlates with the observations by microscope, the crystals that are mainly aggregated (drug-PVP) have the best flowing properties; while ibuprofen crystallized without additives shows the worst flow properties. Drug-agar powder takes an intermediate position. These results can be attributed to particle size of samples. The drug –PVP co-precipitates had superior flow due to larger particle size and near spherical shape (Figures. 1d and e). The area of contacts in the powder bed for spherical shapes was smaller than that for others and this might lead to better flowability. Results of this study confirmed previous works which have shown that differences in crystal habit may strongly influence the particle orientation and modify the flowability and packing characteristics of a drug powder (22). 

*FT-IR, X-ray and DSC*

FT-IR spectra of all samples showed characteristic peaks of ibuprofen at 1720 cm-1(C stretching) and 2955 cm-1 (bonded –OH stretching) indicating that there was no any interactions between the drug and the additives. Change in the relative intensities of XRPD peaks of co-precipitate samples compared with pure drug can be interpreted as follows. Garekani *et al *(23) have attributed decrease in the intensity of XRPD peaks to the changes in crystal habit of drug crystals. As a result of changing crystal habit, the relative abundance of the planes exposed to the X-ray source would have been altered, producing the variations in the relative intensities of the peaks. On the other hand, it has been shown the crystal size can have influence on the intensities of XRPD peaks (24). Change in size and habit of crystals of the co-precipitates in comparison with crystals of pure drug can be seen in Fig. 1. Moreover, decrease in the intensity of XRPD peaks may be due to presence of amorphous regions in the crystals, or due to weakening and disruption of crystal lattice and order. 

The little changes in DSC data of co-precipitate samples compared with pure drug, may be attributed to the presence of amorphous regions in the crystals, or due to weakening and disruption of crystal lattice and order, or may be an effect of crystal size (25). Results from X-ray diffraction analysis and DSC ruled out polymorphic modification.

*Tabletability*

Tabletability is the capacity of a powdered material to be transformed into a tablet of specified strength under the effect of compaction pressure and is represented by a plot of tablet tensile strength against compaction pressure (26). Poor tabletability of ibuprofen crystals can be attributed to the presence of crystal faces that give poor adhesion to other crystals and the absence of the faces that are required for optimal adhesion (27). The presence of polymers (agar and PVP) with ibuprofen crystals in co-precipitated samples affected the properties of their compacts. The increase in tablet strength is influenced by the properties associated with both the polymer and the drug crystals. When drug is co-precipitated with a polymer, the particles will be covered by the polymer which could act as binder. Addition of a binder to a compound generally resulted in an increase in tablet strength. According to previous researches, the addition of binder is normally expected to increase the tensile strength of a pharmaceutical compact compared with compacts of the pure material (28-30). Normally, the binder covering the drug particles increases and changes the nature of the surface area available for interparticulate bonds (28) and the available polymers can act as contact point between the ibuprofen particles. Therefore, both increased total contact points and effective bonding area increase the tablet tensile strength. It has been suggested that the tensile strength of the mixture may best be increased by ensuring a high degree of coverage of the particles by the binder (28) which could be expected to achieve by co-precipitation technique. On the other hand, compaction of the powders into tablets showed that for the materials which fragmented to a limited degree during compression, the particle size and shape affected the compact strength (31-33). However, for materials which fragment markedly during compression, the size and shape of the particles before compaction did not affect compact strength. It has been previously shown that various ibuprofen crystals undergo plastic deformation (low fragmentation) during compression (34, 35). Therefore, according to these facts, improved in tensile strength of the co-precipitated samples may also be attributed to change in crystal shape and size of the ibuprofen crystals. Two –way analysis of variance showed that there were significant differences (*P<*0.05) between the tensile strengths of tablets made from different co-precipitated samples at compression pressures of 25 and 50 MPa. The samples obtained with PVP exhibited the best compression properties, and at each compression force, the tablets had higher tensile strength than tablets made from drug –agar co-precipitates. 

*Dissolution*

According to solubility results, modifying dissolution rate of drug with additive by affecting on solubility of drug was ruled out. The presence of hydrophilic polymers decreased the contact angle of these particles (Table 1). Therefore, the enhancement of the dissolution rate of drug in drug-agar co-precipitates can be attributed to the presence of this hydrophilic polymer, which increases wettability of sample. On the other hand, these data can also be attributed to the smaller particle size of ibuprofen crystals obtained with agar compared with the drug crystals obtained without additives.

However, the amount of drug released is markedly reduced by the presence of PVP (0.5% and 1%) and value even lower than those of ibuprofen alone obtained even though the powder had higher wettability (lower contact angle). Thus, a better wettability caused by PVP in the final product does not cause the effect. Aggregation of drug crystals and consequently larger particle size in drug-PVP co-precipitates may be the main factor that reduces dissolution rate of drug in comparison with other samples.

Moreover, lower dissolution rate of drug –PVP co-precipitates may be attributed to the increase in the thickness of the diffusion layer due to the high viscosity of the polymer (36). PVP does not show saturation solubility as such, but rather swell and absorb water to produce a continuum of concentration between the solid surface and the bulk medium (37). Once in solution in the diffusion layer, the viscosity is sufficiently high to render diffusion through the concentrated layer slow, thereby impeding dissolution. These finding are similar to results of the previous study, where in PVP was found to retard dissolution of mebendazol recrystallized in presence of the polymer (38).

No significant difference (*P>*0.05) could be observed in the percent of drug release from the co-precipitates obtained in presence of 0.1% additives (PVP or agar) compared to the drug crystals obtained without additives. To prevent confusion in dissolution profiles, these results were not reported. 

## Conclusion

The presence of small amount of additive in the co-precipitated samples modified significantly the drug crystal properties. The crystal shape changed from acicular without additive to platy form with agar. While the spherical particles obtained in the presence of PVP seem to be aggregates of numerous fine microcrystals which had stuck together.

Crystals generated using co-precipitation approach in presence of additives were isomorphic with untreated ibuprofen, although they exhibited variable crystal habit and size. While faster drug release was obtained from drug-agar system, those of the drug-PVP system gave slower drug release than crystals grown in the absence of additive. The presence of additives in co-precipitated samples affected the properties of their compacts. The compact tensile strength increased in presence of additives and these were concentration dependent. The co-precipitation technique used is simple and minimizes the use of additives. These co-precipitates may be useful for the preparation of ibuprofen tablets by direct compression method.
